# Investigation of SOI Raman Lasers for Mid-Infrared Gas Sensing

**DOI:** 10.3390/s91007814

**Published:** 2009-09-30

**Authors:** Vittorio M.N. Passaro, Francesco De Leonardis

**Affiliations:** 1 Dipartimento di Elettrotecnica ed Elettronica, Politecnico di Bari, via Edoardo Orabona n. 4, 70125 Bari, Italy; 2 Dipartimento di Ingegneria dell'Ambiente e per lo Sviluppo Sostenibile, Politecnico di Bari, viale del Turismo n. 8, 74100 Taranto, Italy; E-Mail: f.deleonardis@poliba.it

**Keywords:** raman lasers, stimulated raman scattering, nonlinear waveguides, optical sensors, integrated optics, silicon-on-insulator

## Abstract

In this paper, the investigation and detailed modeling of a cascaded Raman laser, operating in the midwave infrared region, is described. The device is based on silicon-on-insulator optical waveguides and a coupled resonant microcavity. Theoretical results are compared with recent experiments, demonstrating a very good agreement. Design criteria are derived for cascaded Raman lasers working as continuous wave light sources to simultaneously sense two types of gases, namely C_2_H_6_ and CO_2_, at a moderate power level of 130 mW.

## 1. Introduction

For over two decades compact, broadly tunable, energy efficient midwave infrared (MWIR) and longwave infrared (LWIR) sources and devices have been the topic of active research [1]. Historically, the need for sources operating especially in the 3–5 μm and 8–12 μm atmospheric transmission windows has been primarily driven by military applications such as wind light detection and ranging (LIDAR), and IR countermeasures (IRCM). However, in recent years such sources have also found use in a wide array of applications ranging from purely scientific uses, such as ring down and Fourier transform infrared (FTIR) spectroscopy, to clinical and industrial uses such as tissue ablation and hydrocarbon detection [2]. In addition, the growing interest for industrial uses such as hydrocarbon detection from vehicle, oil fields, and industrial smoke stacks has recently induced the research to increase its efforts to optimise and study lasers for mid infrared gas sensing.

Laser-based gas sensing is attractive because it can provide a way to achieve highly sensitive, real-time, *in situ* detection of various gases. The key to its success is the development of compact, reliable, and cost-effective laser light sources operating in the infrared region, where various gases exhibit a large number of strong absorption lines originating from fundamental, rotational and vibrational absorption [3,4]. Currently, near-IR distributed feedback (DFB) laser diodes developed for telecommunications are used for gas sensing because they satisfy all these requirements. However, there is a strong need to develop mid-IR laser sources because the absorption intensities of most gases are well larger in the mid-IR than near-IR by a factor 100–10,000. As shown in [3], a large number of very important gases for industrial, environmental and safety needs, such as HCl, CH_4_, C_2_H_6_, CO_2_, NH_3_, N_2_O, SO_2_, H_2_O, can be detected using lasers operating in the mid-IR wavelength range 2–10 μm. Thus, the use of mid-IR lasers is expected to greatly increase the sensitivity of gas sensing and reduce the optical path length and system sizes.

Recently, mid-IR laser sources based on difference frequency generation in quasi-phase-matched (QPM) LiNbO_3_ have been studied as promising candidates for these applications because they can provide continuous wave (CW) mid-IR light in the wavelength range 2–5 μm at room temperature. However, in spite of their excellent sensitivity, the low conversion efficiency of the conventional QPM-LiNbO_3_ devices has limited their use because large, expansive, high power lasers must be also used to achieve a reasonable amount of mid-IR optical output [3]. Moreover, different structures of 3.5 μm small wavelength distributed feedback quantum cascade lasers have actually been developing to achieve low threshold mid-IR sources for gas sensing [5]. A recent work proposed in [6] has shown the possibility to use an interband cascade laser as light source to detect gases in the range λ = 3.6 ÷ 4.3 μm.

Another approach recently proposed to realise mid-IR light sources is based on Stimulated Raman Scattering (SRS) effect. In fact, one of the major advantages of Raman lasers is their ability to generate coherent light in wavelength regions that are not easily accessible with other types of lasers [7]. To this aim, silicon is a particularly suitable material for Raman lasers operating in the near and mid-IR regions because it can guarantee a very good trade-off between low cost and high performance. As well described in [8,9], silicon represents the ideal platform for Integrated Optics and Optoelectronics because the quality of commercial silicon wafers driven by Microelectronics industry continues to improve while the cost continues to decrease. Moreover, the compatibility with silicon integrated circuits manufacturing and silicon Micro-Electro-Mechanical Systems (MEMS) technology is another important reason for this interest in Silicon Photonics. As a transmission medium, silicon has much higher nonlinear effects than the commonly used silicon dioxide, in particular the Raman effect. In fact, Raman gain has been successfully exploited in fiber amplifiers and lasers, but usually several kilometres of fibre are required to create a useful device. Fortunately, the gain coefficient for SRS is approximately four orders of magnitude larger in silicon than in silica. Additionally, silicon-on-insulator (SOI) waveguides can confine the optical field to an area that is approximately 100 times smaller than the modal area in a standard single-mode optical fiber. These two circumstances allow the fabrication of efficient Raman-based centimeter-scale integrated optical devices to be achieved. Starting from 2002, several experimental and theoretical studies based on this effect have been proposed in literature, such as Raman amplification in SOI waveguides [10-15], Stokes and anti-Stokes Raman conversion [16-19], cross phase modulation-based interferometer switch [20] two-photon absorption [21,22], lossless modulation [23] to point-out the real efficiency to use SRS effect on the Silicon platform. More recently, efficient Raman lasing in silicon in near-IR (NIR) region has been experimentally and theoretically demonstrated [24-28], showing great potential for realising low-cost, compact, room-temperature lasers in MWIR [1,29-33].

Therefore, in this work we theoretically analyze the possibility to realise a Raman cascaded laser for simultaneously sensing two different gases having their absorption peaks in the wavelength range 3–5 μm. Our choice is motivated by two concurrent aspects. First, the cascade Raman emission can result attractive in the range 3–5 μm, since two photon absorption (TPA) and free carrier absorption (FCA) effects are completely eliminated. Second, the cascade Raman laser could represent an efficient answer to the drawbacks of interband diode lasers and quantum cascade lasers. In fact, the ability to induce the cascaded lasing by means of SRS effect can potentially produce several wavelengths in the range 2–5 μm, at the same time too long for interband diode lasers to be reached due to Auger recombination, and usually too short for quantum cascaded lasers owing to the finite-conduction-band offset at room temperature [34,35]. However, two works have also demonstrated the possibility to realise light sources by means of interband diode lasers and quantum cascaded lasers emitting around 3.8 μm [6,36].

This paper is organized as follows. In Section 2 we derive the mathematical model to study the nonlinear effects in a resonant microcavity coupled to an external waveguide, following a different approach from literature [30]. The proposed modeling includes all nonlinear effects involved in the integrated structure without any a-priori assumption, including SRS, Self-Phase-Modulation (SPM) and Cross-Phase-Modulation (XPM) effects as induced by Kerr nonlinearity, nonlinear birefringence effect as induced by the modal birefringence and walk-off. The model considers the interaction between pump pulse and first-order Stokes wave, as well as excitation of higher-order Stokes waves, mismatch between the input beams and microcavity resonance wavelengths, and coupling mechanism between microcavity and bus waveguide. In Section 3 a number of numerical results are presented, including comparisons between our theory and some experiments in literature on CW microcavity-based cascaded Raman lasers for validating our approach. Moreover, a systematic study of SOI waveguides modal properties, the polarization dependence of coupling factors of both pump and Stokes waves and the laser performance are investigated at MWIR for the first time, to the best of our knowledge. Finally, Section 4 summarizes the conclusions.

## 2. Device Modeling

In this section, a very accurate physical model proposed in our previous works [37-39] is generalized to analyse the Raman lasing effect in a SOI microcavity resonator working at mid-IR. The model is based on a set of partial differential equations for nonlinear coupling among pump, first-order and higher-order Stokes waves inside the microcavity, considering both polarization states.

Modeling of cascaded Raman laser in mid-IR region has been already proposed in literature [30]. However, differently from that approach where the source is obtained by placing the silicon waveguide between two highly reflecting elements such as Bragg gratings or dielectric coatings, we consider the racetrack resonator coupled externally with the bus waveguide as laser architecture. This fundamental difference induces to extend the model in [30] to include in the physical analysis the resonance condition mismatch of input pump and Stokes waves as originated inside the resonator, as well as the effect of photon decay time induced by the coupling process. In addition, differently from other experimental and theoretical works presented in literature [30-32], we also consider in this paper as the waveguide size influences the modal birefringence, the group velocity mismatch and, thus, the laser performance.

**Figure 1.**
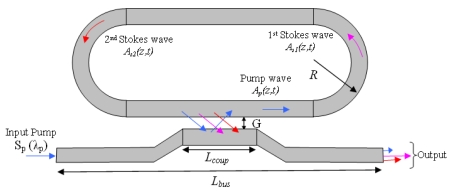
Schematic architecture of a cascaded racetrack-resonator SOI Raman laser.

In our analysis we assume the architecture as sketched in Figure 1, where the input pump (*S_p_*) is injected in the resonant microcavity by evanescent coupling from an external SOI bus waveguide. As it was already experimentally demonstrated [26,27,31], the most efficient architecture for the laser cavity is represented by a racetrack resonator. In fact, it leads us to control the proper combination of both pump and Stokes coupling factors by changing the coupling length *L_coup_*, gap *G* and polarization state.

The scheme of Figure 1 also clarifies the concept of a cascaded racetrack-resonator SOI Raman laser. The pump coupled into the laser cavity generates optical gain by SRS inside the silicon waveguide at the first-order Stokes wavelength, which is 15.6 THz red-shifted from the pump. The gain increases with the pump power, and lasing threshold is reached when the optical gain equals the total cavity loss. The optical power of the first-order Stokes wave starts to increase inside the cavity, inducing a pump depletion and eventually gain for the second-order Stokes signal. This cascaded process can continue and generate higher-order Stokes lasing at longer wavelengths. It is worth to outline that the cascaded racetrack-resonator SOI Raman laser shown in Figure 1 represents the laser head for the gas detection equipment. In fact, an experimental setup to demonstrate the gas detection should involve driver, laser head, filters, gas cells, photodetectors and electronic system for signal processing [3]. Thus, the input pump laser modulated by the driver produces the mid-IR outputs as described above, that are collimated by means of filters into the gas cells and detected by photodetectors. Finally, the photocurrents are processed by the electronic circuit.

**Figure 2.**
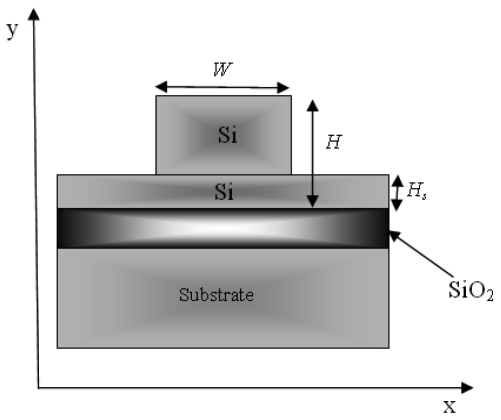
Cross-section of SOI waveguide.

Hereinafter, we assume a SOI waveguide as sketched in Figure 2, having rib total height *H*, slab height *H_s_* and rib width *W*. Without any lack of generality, the electric field inside the microcavity is predominantly assumed as a single transverse mode. This condition can be satisfied by choosing appropriate rib sizes to meet the single-mode condition.

In a single-mode SOI waveguide two propagating modes are typically confined, one quasi-TE (dominant x-component of electric field) and one quasi-TM (dominant y-component). Our coupled-mode approach describes the power transfer among pump wave (*p*), first-order Stokes wave (*s*_1_) and higher-order Stokes waves. Two higher-order Stokes waves (*s*_2_,*s*_3_) and both polarizations are considered.

Under assumption of translational invariance along the propagation direction (z), due to large radius *R* of racetrack resonator (see Figure 1), the electric field in the single mode SOI waveguide can be written using the variable separation principle as E(*x,y,z,t*) = *C F* (*x,y*) *A*(*z,t*) e^jk̄z^where *k̄* is the propagation constant and *F*(*x,y*) is the optical mode distribution in the waveguide cross section x − y (solutions of Helmholtz wave equation), *C* is a normalization constant, $C=1/\sqrt{\int \int_{-\infty }^{+\infty }{\left|F\left(\text{x},\text{y}\right)\right|}^{2}\text{dxdy}}$ and *A*(*z,t*) is the slowly-varying wave amplitude. Thus, without any leak of generality, the total electric field inside the SOI waveguide can be written as:

(1) $$\begin{array}{l} \text{E}\left(x,y,z,t\right)=\hat{\text{x}}\left[\begin{array}{l} \sum_{m}C_{1,m}A_{1,m}(z,t)F_{1,m}(x,y)e^{j\left({\bar{k}}_{1,m}z-\omega_{p,m}t\right)}+\sum_{n}C_{3,n}A_{3,n}(z,t)F_{3,n}(x,y)e^{j\left({\bar{k}}_{3,n}z-\omega_{s1,n}t\right)}\\ \sum_{r}C_{5,r}A_{5,r}(z,t)F_{5,r}(x,y)e^{j\left({\bar{k}}_{5,r}z-\omega_{s2,r}t\right)}+\sum_{q}C_{7,q}A_{7,q}(z,t)F_{7,q}(x,y)e^{j\left({\bar{k}}_{7,q}z-\omega_{s3,q}t\right)} \end{array}\right]\\ \;+\hat{\text{y}}\left[\begin{array}{l} \sum_{m}C_{2,m}A_{2,m}(z,t)F_{2,m}(x,y)e^{j\left({\bar{k}}_{2,m}z-\omega_{p,m}t\right)}+\sum_{n}C_{4,n}A_{4,n}(z,t)F_{4,n}(x,y)e^{j\left({\bar{k}}_{4,n}z-\omega_{s1,n}t\right)}\\ \sum_{r}C_{6,r}A_{6,r}(z,t)F_{6,r}(x,y)e^{j\left({\bar{k}}_{6,r}z-\omega_{s2,r}t\right)}+\sum_{q}C_{8,q}A_{8,q}(z,t)F_{8,q}(x,y)e^{j\left({\bar{k}}_{8,q}z-\omega_{s3,q}t\right)} \end{array}\right] \end{array}$$

(where the meaning of superscripts and subscripts is as follows: 1 = *p*^(^*^TE^*^)^, 2 = *p*^(^*^TM^*^)^, 3 = s_1_^(^*^TE^*^)^, 4 = *s*_1_^(^*^TM^*^)^ 5 = *s*_2_^(^*^TE^*^)^ 6 = *s*_2_^(^*^TM^*^)^ 7 = *s*_3_^(^*^TE^*^)^ 8 = s_3_^(^*^TM^*^)^ In this equation, summations in **x** ^ and **y** ^ are relevant to quasi-TE and quasi-TM modes, respectively, subscripts *m, n, r* and *q* designate all possible cavity longitudinal modes for pump and first, second and third Stokes waves, respectively, *ω_p_*_,_*_m_* (*ω_s1_*_,_*_n_, ω_s2_*_,_*_r_, ω_s3_*_,_*_q_*) is the resonant angular pulsation of the pump (Stokes) mode inside the cavity and *k̄_i,m_* (*i* = 1, 2) is the pump propagation constant under resonance condition.

As detailed in [38], it is clear that only a few terms are to be considered in the summations of Equation (1) under external excitation. In fact, if the input pump (*S_p_*)is launched in the bus waveguide with angular frequencies *ω_p_*, the first-order Stokes wave is originated at *ω_s_= ω_p_* − Ω*_R_*, being Ω*_R_* = 15.6 THz the Raman frequency shift in silicon. In this condition, only those modes whose resonant angular frequencies are closer to *ω_p_* and *ω_s1_* will give a contribution to Equation (1). Similar considerations hold for higher-order Stokes waves. These modes are characterized by an longitudinal order, namely, *m̄ n̄, r̄*, and *q̄*, evaluated by means of the resonance condition as, *m̄* ∼ *ω_p_n_eff_,_p_L_Cavity_/*(*2*πc), *n̄* ∼ *ω_s_*_1_ *n_eff_,_s_*_1_ *L_Cavity_/*(*2*πc), *r̄* ∼ *ω_s_*_2_ *n_eff_,_s_*_2_ *L_Cavity_/*(*2*πc), and *q̄* ∼ *ω_s_*_3_ *n_eff_,_s_*_3_ *L_Cavity_/*(*2*πc), being *n_eff_*_,_*_p_*(*n_eff_*_,_*_s_*_1_, *n_eff_*_,_*_s_*_2_, *n_eff_*_,_*_s_*_3_) the effective index of pump (Stokes) wave inside the racetrack resonator, c the light velocity in vacuum, and *ω_p_, ω_s1_, ω_s2_, ω_s3_* the angular frequencies of pump, first-order, second order and third-order Stokes waves, respectively.

Moreover, the previous consideration holds if the cavity free spectral range (FSR) is larger than the input pulse bandwidth, δ*ω_pulse_*. For example, assuming a Gaussian input pulse with full-wave-half-maximum (FWHM) time width *T_FWHM_*, the condition *FSR* > δ*ω_pluse_* (pulse bandwidth) gives $L_{\text{cavity}}<\left(2\pi cT_{\text{FWHM}}\right)/\left(2\sqrt{2\ln2}n_{\text{eff},p}\right)$, being *L_cavity_* the cavity perimeter [38]. Then, by assuming *T_FWHM_* = 100 ps, the cavity length required to consider only two resonant modes for the pump wave and aligned as quasi-TE and quasi-TM modes can be estimated to be smaller than 2.8 cm, that is a reasonable condition in some integrated structures. Now, by following the procedure outlined in [37,38] and assuming the nonlinear contributions to **P***_NL_* as a small perturbation of refractive index, we have obtained the following coupled equations for pump waves:

(2) $$\begin{array}{l} v_{g,\xi }\frac{\partial A_{\xi }}{\partial z}+\frac{\partial A_{\xi }}{\partial t}+j\frac{1}{2}v_{g,\xi }\beta_{2,\xi }\frac{\partial^{2}A_{\xi }}{\partial t^{2}}-\frac{1}{6}v_{g,\xi }\beta_{3,\xi }\frac{\partial^{3}A_{\xi }}{\partial t^{3}}=j\left(\omega_{p,\bar{m}}-\omega_{p}\right)A_{\xi }-\frac{1}{2}\frac{1}{\tau_{\xi }}A_{\xi }+jv_{g,\xi }\gamma_{\xi ,\xi }{\left|A_{\xi }\right|}^{2}A_{\xi }\\ +j2v_{g,\xi }\gamma_{\xi ,\upsilon }{\left|A_{\upsilon }\right|}^{2}A_{\xi }+j2v_{g,\xi }\gamma_{\xi ,3}{\left|A_{3}\right|}^{2}A_{\xi }+j2v_{g,\xi }\gamma_{\xi ,4}{\left|A_{4}\right|}^{2}A_{\xi }+2jv_{g,\xi }k_{\xi ,3,\upsilon ,4}A_{\upsilon }A_{3}^{*}A_{4}e^{j({\bar{k}}_{\upsilon }+{\bar{k}}_{4}-{\bar{k}}_{\xi }-{\bar{k}}_{3})z}\\ +2jv_{g,\xi }k_{\xi ,4,\upsilon ,3}A_{\upsilon }A_{4}^{*}A_{3}e^{j({\bar{k}}_{\upsilon }+{\bar{k}}_{3}-{\bar{k}}_{\xi }-{\bar{k}}_{4})z}+jv_{g,\xi }k_{\xi ,\xi ,\upsilon ,\upsilon }A_{\upsilon }A_{\xi }^{*}A_{\upsilon }e^{j(2{\bar{k}}_{\upsilon }-2{\bar{k}}_{,\xi })z}\\ -\frac{1}{2}v_{g,\xi }g_{R}\frac{\omega_{p}}{\omega_{s1}}f_{\xi ,3}{\left|A_{3}\right|}^{2}A_{\xi }-\frac{1}{2}v_{g,\xi }g_{R}\frac{\omega_{p}}{\omega_{s1}}f_{\xi ,4}{\left|A_{4}\right|}^{2}A_{\xi }+\frac{\eta_{\xi }}{L_{\text{cavity}}}v_{g,\xi }S_{\xi }\\ \xi =1,2 \end{array}$$

being *ζ* = 1,2 for quasi-TE and quasi-TM polarization, respectively.

Then, we have for quasi-TE and quasi-TM first (*κ* = 3,4) and second order Stokes (*κ* = 5,6) pulses:

(3) $$\begin{array}{l} v_{g,\kappa }\frac{\partial A_{\kappa }}{\partial z}+\frac{\partial A_{\kappa }}{\partial t}+jv_{g,\kappa }\frac{1}{2}\beta_{2,\kappa }\frac{\partial^{2}A_{\kappa }}{\partial t^{2}}-v_{g,\kappa }\frac{1}{6}\beta_{3,\kappa }\frac{\partial^{3}A_{\kappa }}{\partial t^{3}}=j\left({\bar{\omega }}_{\kappa }-\omega_{\kappa }\right)A_{\kappa }-\frac{1}{2}\frac{1}{\tau_{\kappa }}A_{\kappa }+j2v_{g,\kappa }\gamma_{\kappa ,\mu }{\left|A_{\mu }\right|}^{2}A_{\kappa }\\ +j2v_{g,\kappa }\gamma_{\kappa ,\mu +1}{\left|A_{\mu +1}\right|}^{2}A_{\kappa }+jv_{g,\kappa }\gamma_{\kappa ,\kappa }{\left|A_{\kappa }\right|}^{2}A_{\kappa }+j2v_{g,\kappa }\gamma_{\kappa ,\nu }{\left|A_{\nu }\right|}^{2}A_{\kappa }+\frac{1}{2}v_{g,\kappa }g_{R,\kappa }f_{\kappa ,\mu }{\left|A_{\mu }\right|}^{2}A_{\kappa }\\ +v_{g,\kappa }\frac{1}{2}g_{R,\kappa }f_{\kappa ,\mu +1}{\left|A_{\mu +1}\right|}^{2}A_{\kappa }+j2v_{g,\kappa }k_{\kappa ,\mu ,\mu +1,\nu }A_{\mu +1}A_{\mu }^{*}A_{\nu }e^{j({\bar{k}}_{\mu +1}+{\bar{k}}_{\nu }-{\bar{k}}_{\mu }-{\bar{k}}_{\kappa })z}+j2v_{g,\kappa }k_{\kappa ,\mu +1,\mu ,\nu }A_{\mu }A_{\mu +1}^{*}A_{\nu }e^{j({\bar{k}}_{\mu }+{\bar{k}}_{\nu }-{\bar{k}}_{\mu +1}-{\bar{k}}_{\kappa })z}\\ +jv_{g,\kappa }k_{\kappa ,\kappa ,\nu ,\nu }A_{\nu }A_{\kappa }^{*}A_{\nu }e^{j(2{\bar{k}}_{\nu }-2{\bar{k}}_{\kappa })z}-\frac{1}{2}v_{g,\kappa }g_{R,\kappa }f_{\kappa ,\mu +4}{\left|A_{\mu +4}\right|}^{2}A_{\kappa }-\frac{1}{2}v_{g,\kappa }g_{R,\kappa }f_{\kappa ,\mu +5}{\left|A_{\mu +5}\right|}^{2}A_{\kappa }\\ +j2v_{g,\kappa }k_{\kappa ,\mu +4,\nu ,\mu +5}A_{\nu }A_{\mu +4}^{*}A_{\mu +5}e^{j({\bar{k}}_{\nu }+{\bar{k}}_{\mu +5}-{\bar{k}}_{\kappa }-{\bar{k}}_{\mu +4})z}+j2v_{g,\kappa }k_{\kappa ,\mu +5,\nu ,\mu +4}A_{\nu }A_{\mu +5}^{*}A_{\mu +4}e^{j({\bar{k}}_{\nu }+{\bar{k}}_{\mu +4}-{\bar{k}}_{\kappa }-{\bar{k}}_{\mu +5})z}\;\\ \kappa =3,4,5,6 \end{array}$$

and for quasi-TE and quasi-TM third order Stokes (*ρ* = 7, 8) waves:

(4) $$\begin{array}{l} v_{g,\rho }\frac{\partial A_{\rho }}{\partial z}+\frac{\partial A_{\rho }}{\partial t}+j\frac{1}{2}v_{g,\rho }\beta_{2\rho }\frac{\partial^{2}A_{\rho }}{\partial t^{2}}-\frac{1}{6}v_{g,\rho }\beta_{3,\rho }\frac{\partial^{3}A_{\rho }}{\partial t^{3}}=j\left(\omega_{s3,\bar{q}}-\omega_{s3}\right)A_{\rho }-\frac{1}{2}\frac{1}{\tau_{\rho }}A_{\rho }\\ +jv_{g,\rho }2\gamma_{\rho ,5}{\left|A_{5}\right|}^{2}A_{\rho }+jv_{g,\rho }\gamma_{\rho ,6}{\left|A_{6}\right|}^{2}A_{\rho }+jv_{g,\rho }\gamma_{\rho ,\rho }{\left|A_{\rho }\right|}^{2}A_{\rho }+j2v_{g,\rho }\gamma_{\rho ,\zeta }{\left|A_{\zeta }\right|}^{2}A_{\rho }\\ +\frac{1}{2}v_{g,\rho }g_{R,3}f_{\rho ,5}{\left|A_{5}\right|}^{2}A_{\rho }+\frac{1}{2}v_{g,\rho }g_{R,3}f_{\rho ,6}{\left|A_{6}\right|}^{2}A_{\rho }+j2v_{g,\rho }k_{\rho ,5,6,\zeta }A_{6}A_{5}^{*}A_{\zeta }e^{j({\bar{k}}_{6}+{\bar{k}}_{\zeta }-{\bar{k}}_{\rho }-{\bar{k}}_{5})z}\\ +j2v_{g,\rho }k_{\rho ,6,5,\zeta }A_{5}A_{6}^{*}A_{\zeta }e^{j({\bar{k}}_{5}+{\bar{k}}_{\zeta }-{\bar{k}}_{6}-{\bar{k}}_{\rho })z}+jv_{g,\rho }k_{\rho ,\rho ,\zeta ,\zeta }A_{\zeta }A_{\rho }^{*}A_{\zeta }e^{j(2{\bar{k}}_{\zeta }-2{\bar{k}}_{\rho })z} \end{array}\rho =7,8$$

In Equation (2) subscript *υ* = 2,1 holds when *ζ* = 1,2, respectively. In Equation (3), if *κ* = 3,4, *ω_k_=ω_s_*_1_, *ω̄_k_*=*_ωs1,n_μ* = 1 and *ν* = 4,3, while if κ=5,6 *ω_k_* = *ω_s2_, ω̄_k_* = *ω_s2*r̄*_ μ* = 3 and *ν* = 6,5. Moreover, $g_{R,\kappa }≃g_{R}\frac{\omega_{\kappa }}{\omega_{s1}}$. In Equation (4), it holds *ζ* = 8,7 when *q* = 7,8 respectively, and $g_{R,3}≃g_{R}\frac{\omega_{s3}}{\omega_{s1}}$ In conclusion, Equation (2) takes into account the time-space evolution of both quasi-TE and quasi-TM pump pulses. Similar evolutions are described by Equation (3) for first and second order Stokes pulses of both polarizations, and by Equation (4) for third order Stokes pulses of both polarizations. In equation system (2)–(4), the asterisk denotes the complex conjugate. The partial differential equation system (2)–(4) clearly represents a generalization for mid-IR laser applications of the model proposed in [37,38]. The terms (*ω_p,m_* − *ω_p_*), (*ω_s_*_1,__*n̄*_ *_,_* − *ω_s_*_1_), (*ω_s_*_2,__*r̄*,_ − *ω_s_*_2_), and (*ω_s_*_3,__*q̄*_*_,_* − *ω_s_*_3_) designate the mismatch from the resonance condition of input pump and Stokes waves originated inside the resonator, respectively. The term *τ_ζ_* with ζ = 1,2 (*τ_κ_, τ_ρ_*) represents the overall photon decay time of the pump (Stokes) wave inside the cavity. It can be expressed as a function of the following two terms:

(5) $$\frac{1}{\tau_{\xi }}=\frac{1}{\tau_{l,\xi }}+\frac{1}{\tau_{c,\xi }}$$

where τ*_l_*_,_*_ζ_*, and τ*_c_*_,_*_ζ_* represent the decay time related to total linear losses inside the cavity and coupling between the optical resonator and external bus waveguide, respectively. As detailed in our previous work [37], these terms can be calculated as:

(6) $$\tau_{l,\xi }=\frac{1}{\left(\alpha_{\text{loss},\xi }^{\text{tot}}v_{g,\xi }\right)}\;\tau_{c,\xi }=\frac{1}{\eta_{\xi }^{2}}\frac{L_{\text{cavity}}}{v_{g,\xi }}$$

being *ν_g,ζ_* the pump wave group velocity, $\alpha_{\text{loss},\xi }^{\text{tot}}$ the overall linear loss coefficient related to propagation, bending and scattering loss contribution, and $\eta_{\xi }^{2}$ the coupling factor, defined as the power fraction of the pump wave leaving the resonator due to the directional coupler. Similar definition holds for *τ_κ_* and *τ_ρ_*.

In Equations (2)–(4), *g_R_* = 3*ω_s_*_1_*μ*_0_*χ^R^*(ω*_R_*)/(*n_eff,p_n_eff,s_*_1_) [2] is the Raman gain with *μ*_0_ the magnetic susceptibility, *n_eff,p_*, and *n_eff,s1_* the effective refractive index of the pump and first-order Stokes waves, respectively, and *χ^R^*(ω*_R_*) the Raman–resonant susceptibility, defined as in [16]. Moreover, +*g_R_* (positive sign) designates the SRS effect, while −*g_R_* (negative sign) determines the depletion effect due to the energy exchange induced by SRS for *n* -th order Stokes wave, if (n + 1)-th order Stokes wave is simultaneously present in the relevant equation.

Moreover, the coefficients *γ_i,i_* = *n*_2_*ω_i_f_i,i_*/*c* and *γ_i,j_* = *n*_2_*ω_i_f_i,j_*/*c* take into account SPM and XPM effects as induced by Kerr nonlinearity, being c the light velocity and *n*_2_ the nonlinear refractive index [39,40]. Terms *k_i,j,k,l_* = *n*_2_*ω_i_f_i,j,k,l_/c, i,j* = 1,…,8, depend on birefringence effects. The overlap integrals *f_i,j_* and *f_i,j,k,l_* are given by:

$$\begin{array}{c} f_{i,j}=\frac{\iint {\left|F_{i}\left(x,y\right)\right|}^{2}{\left|F_{j}\left(x,y\right)\right|}^{2}\text{dxdy}}{\iint {\left|F_{i}\left(x,y\right)\right|}^{2}\text{dxdy}\iint {\left|F_{j}\left(x,y\right)\right|}^{2}\text{dxdy}}i,j=1,\ldots ,8\\ f_{i,j,k,l}=\frac{\int \int_{-\infty }^{+\infty }F_{i}^{*}F_{j}^{*}F_{k}F_{l}\text{dxdy}}{\sqrt{\left(\int \int_{-\infty }^{+\infty }{\left|F_{i}\right|}^{2}\text{dxdy}\right)\left(\int \int_{-\infty }^{+\infty }{\left|F_{j}\right|}^{2}\text{dxdy}\right)\left(\int \int_{-\infty }^{+\infty }{\left|F_{k}\right|}^{2}\text{dxdy}\right)\left(\int \int_{-\infty }^{+\infty }{\left|F_{l}\right|}^{2}\text{dxdy}\right)}}i,j,k,l=1,\ldots ,8 \end{array}$$

In particular, $f_{i,i}^{-1}=A_{\text{eff},i}$ represents the effective core area of the optical mode relevant to the *i*-th pulse, i = 1,…,8. Terms *k_i,i,j,j_* are usually dominant (*k_i,i,j,j_* > *k_i,j,k,l_* for *i* ≠ *j, k* ≠ *l*) and represent the coherent coupling between the polarization components for waves at the same frequency, giving degenerate four-wave mixing [41]. Rigorously, additional terms should be introduced in Equation (4) to take into account XPM and FWM effects between third-order Stokes waves and pump waves.

However, since these terms can be considered as negligible they are not included in Equation (4). In fact, we can assume FWM effect between third-order Stokes waves and pump waves as negligible due to a too large phase mismatch, while the XPM effect between the third-order Stokes waves and the pump waves can be considered negligible with respect to XPM effect between third-order and second-order Stokes waves, due to the strong pump depletion effect induced by the first-order Stokes wave. In addition, in continuous-wave regime all terms related to SPM and XPM effects do not influence strongly the laser behavior since Raman susceptibility dominates over Kerr susceptibility in SOI technology.

For completeness, group velocity dispersion (GVD) and third-order dispersion (TOD) effects are also included in the model, as indicated by the terms proportional to *β*_2_*_,i_* and *β*_3_*_,i_*. In any case, these effects are removed from physical description in case of CW Raman laser applications. On the contrary, with pulsed Raman lasers GVD and TOD effects are not negligible for very short pulses. However, in this case, input pump pulse *T_FWHM_* is much shorter than *τ_c,ζ_*, then the pump energy inside the resonator cannot be enhanced. Thus, as demonstrated in [38], pulsed excitation in resonator-based Raman lasers should require *T_FWHM_* ≫ *τ_c,ζ_* to induce the SRS effect above the threshold.

Finally, it is worth to note that TPA and FCA effects are not included in our equations due to excellent transmission of silicon in mid IR because of the absence of TPA for wavelengths longer than 2.2 μm [2]. Thus, cascade Raman emission can result very attractive in the range 3–5 μm, since TPA and FCA effects are completely absent.

## 3. Numerical Results and Discussion

### 3.1. Comparisons with Experimental Measurements

To test the analytical formulas and physical assumptions for Raman effect into optical microcavities under CW operation, we have compared our numerical results with some experiments proposed in literature. A very interesting set of comparisons with experimental results involves the CW cascaded Raman laser based on SOI resonator. The architecture used in the experimental setup [31] is the same as Figure 1, where the microcavity is constituted by a SOI racetrack resonator working with a pump beam at 1.55 μm. The silicon waveguide cavity used in the experiment was fabricated on a SOI wafer with a 1 μm buried oxide layer, using CMOS compatible fabrication processes. The proposed device has rib width *W* = 1.5 μm, height *H* = 1.55 μm, and etch depth 0.7 μm. The coupler gap is 0.7 μm, and the coupler length ranges between 900 and 1,100 μm to obtain the desired coupling coefficients for pump and signal wavelengths. The total length of racetrack cavity is 3 cm and the bend radius is 400 μm. The bus waveguide is 1.6 cm long and it is connected to the ring cavity by means of the directional coupler. Finally, the measured optical parameters include *α_loss_* = 0.2 ± 0.05 dB/cm, *τ_eff_* = 0.4 ns, *g_R_* = 10.5 cm/GW, and *β^TPA^* = 0.5 cm/GW, being *β^TPA^* the two-photon absorption coefficient. In order to achieve cascaded lasing, it is crucial to design and fabricate the directional coupler to have the proper combination of coupling factors for pump and Stokes waves. To this aim, we have assumed the same coupler geometry as in [31] and the relevant coupling factors have been evaluated by means of a 3D CAD tool based on beam propagation method (BPM) [42]. Thus, our simulations are not conditioned by any arbitrary choice or fitting parameters.

It is very important to consider also TPA and FCA effects in Equations (2)–(4), since the laser pump is tuned at 1.55 μm. Thus, according to our previous work [37], we include the contribution $\alpha_{i}^{\left(\text{FCA}\right)}$, *i* = 1,2,…8, to the total losses, as induced by free carrier density change generated mainly by TPA of pump pulse. In addition, $\alpha_{i}^{\left(\text{FCA}\right)}$ is evaluated according to Soref's relationship [43] as:

(7) $$\alpha_{i}^{\left(\text{FCA}\right)}=8.5\cdot 10^{-18}\cdot {\left(\frac{\lambda_{i}}{1.55}\right)}^{2}\Delta N_{e}+6.0\cdot 10^{-18}\cdot {\left(\frac{\lambda_{i}}{1.55}\right)}^{2}\Delta N_{h}=\sigma_{i}\cdot N_{c}=\sigma_{0}\cdot {\left(\frac{\lambda_{i}}{1.55}\right)}^{2}N_{c}$$

where *N_c_* = Δ*N_e_* = Δ*N_h_* is the density of electron-hole pairs generated by TPA process. The coefficient *σ*_0_ = 1.45 × 10^−17^ cm^−2^ [16] is the FCA cross section measured at *λ* = 1.55 μm, and *λ_i_* is the relevant mode wavelength (either pump or Stokes wave). Finally, the rate equation governing the free carrier dynamics into the waveguide core is required together with system (2)-(4), given by [12]:

(8) $$\frac{dN_{c}}{dt}=-\frac{N_{c}}{\tau_{\text{eff}}}+\frac{\beta^{TPA}}{2\hbar \omega_{p}}{\left({\left|A_{\xi ,\xi }(z,t)\right|}^{2}f_{\xi ,\xi }\right)}^{2}$$

where *τ_eff_* is the relevant effective recombination lifetime for free carriers, *ℏ︀* is the reduced Planck constant, and *β^TPA^* is the coefficient of carrier generation induced by TPA process of the pump beam. In turn, this nonlinear absorption effect requires the inclusion of contributions 0.5ν*_g,ζ_β^TPA^f_ζ,ζ_*‖*A_ζ_*‖^2^*A_ζ_*, ν*_g,κ_β^TPA^f_κ,κ_*‖*A_ζ_*‖^2^*A_κ_*, and ν*_g,ρ_β^TPA^f_ρ,ρ_*‖*A_ζ_*‖^2^*A_ρ_* in Equations (2), (3) and (4), respectively.

**Figure 3.**
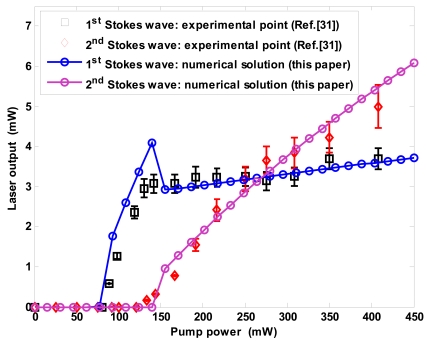
Comparison between experimental data in literature and modeling of this paper in terms of CW cascaded Raman emission versus input pump power (*λ_p_* = 1.55 μm, *λ_s_*_1_ = 1.686 μm, *λ_s_*_2_ = 1.848 μm).

Figure 3 shows the laser output power (Stokes power at the bus waveguide end) versus pump input power. The black and red markers represent the experimental data for first and second order Stokes waves, respectively. In experiment, the pump wavelength is at 1,550 nm and the first and second lasing wavelengths are measured at 1,686 nm and 1,848 nm, respectively.

The solid lines designate our numerical results as evaluated by solving the coupled equations proposed in the model. Our numerical calculations demonstrate the same group velocity inside the resonator for pump and Stokes waves, and thus the partial differential equations (2)–(4) can be transformed in ordinary differential equations by simply writing [38]:

(9) $$\frac{dA_{p,s}}{dt}=\frac{\partial A_{p,s}}{\partial t}+v_{g}\frac{\partial A_{p,s}}{\partial z}$$

where ν*_g,p_* ≅ ν*_g,s_*_1_ ≅ ν*_g, s_*_2_ = ν*_g_* = 8.2645 × 10^7^ m/s. The plots show a very good agreement with experimental data in terms of threshold values, output powers above threshold, external efficiencies and output saturation. In particular, the good agreement with first Stokes output saturation confirms how the Raman cascaded lasing is well described by the mathematical model proposed in this paper.

### 3.2. Waveguide Optical Properties in Mid-IR

To the best of our knowledge, systematic investigations have been not yet presented in literature for optical properties of SOI waveguides in mid-IR region, although this is a topic of increasing interest [44]. The goal of this sub-section is to determine the MWIR optical characteristics of SOI waveguides in terms of birefringence and group velocity mismatch.

As it is well known, a single-mode waveguide can in general support two modes polarized in orthogonal directions. Under ideal conditions, a mode excited with its dominant polarization, i.e., in x direction (quasi-TE mode), would not coupled to the mode with orthogonal dominant y-polarization state (quasi-TM). However, in real waveguides random variations of cross section shape and stress-induced anisotropy result in a mixing of two polarization states. Thus, the two modes exchange their powers in a periodic fashion as they propagate inside the waveguides with period $L_{B}=\lambda /\left(\left|n_{\text{eff}}^{\text{TM}}-n_{\text{eff}}^{\text{TM}}\right|\right)$. It is worth to note that the modal birefringence could induce the nonlinear birefringence [39], as considered in the model through coefficients *k_i,j,k,l_*. In fact, since the electric field associated with an arbitrarily polarized optical wave can be written as in Equation (1), the nonlinear part of induced polarization can be given in similar form but each component depending on third-order susceptibility (Kerr effect) [41]. In fact, since the third-order nonlinearity involves in general interaction among four optical waves, following the algebra details in [41] each component of the induced polarization nonlinear part can be given as the sum of a number of terms proportional to *n*_2_ and to the products between four electric field components. Some of these terms are responsible for SPM and XPM effects, while the remaining terms in *k_i,j,k,l_* are related to FWM. In addition, as explained in [41], significant FWM effect occurs only if the phase mismatch nearly vanishes. This should require matching of both frequencies and wave vectors. In case of CW Raman laser applications, the nonlinear birefringence could become evident as a rotation of the polarization ellipse [41]. This effect can be neglected by appropriately choosing the cavity length.

**Figure 4.**
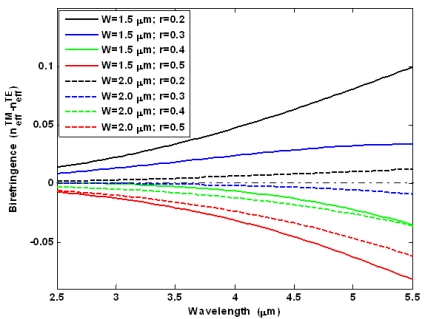
Modal birefringence spectra for different values of *r* and *W*.

Figure 4 shows the modal birefringence $(\Delta n_{\text{eff}}=n_{\text{eff}}^{\text{TM}}-n_{\text{eff}}^{\text{TE}})$ spectra for different values of *r* = *H_s_*/*H*. In particular, in our simulations we have assumed a rib total height *H* = 2.2 μm, and a rib width *W* of 1.5 μm (solid lines) and 2 μm (dashed lines). Hereinafter, calculations of waveguide optical properties in mid-IR region have been carried out by full-vectorial finite element method (FEM) [45].

The plots show that for each value of the rib width, the modal birefringence changes sign with increasing *r* from 0.2 to 0.5, increasing with wavelength. In addition, *W*= 2 μm leads a reduced value of ‖Δ*n_eff_*‖ for each value of *r* and in all wavelength ranges to be achieved. The curves of Figure 4 are useful to calculate the beat length *L_B_* and thus the minimum cavity length needful to avoid the nonlinear birefringence effect. In fact, it occurs *L_cavity_*≫*L_B_* for long waveguides with large birefringence, then the terms containing *k_i,j,k,l_* often change sign and the total phase contribution due to birefringence averages out to zero [39]. In contrast, this contribution is not negligible if *L_cavity_* ≤ *L_B_* (*k_i,i,j,j_*), as it occurs in short waveguides with moderate birefringence. Using the simulations proposed in Figure 4, we can observe that for *W* = 1.5 μm, *r* = 0.2 and operating wavelength 5.5 μm, the modal birefringence has maximum value, inducing a beat length of *L_B_* = 55 μm. Thus, a cavity length much larger than 55 μm should be required to average to zero the contributes depending on *k_i,j,k,l_*. In particular, it is very interesting the case with *W* = 2 μm and *r* = 0.3, where the curve shape is flat around zero in 2.5–3.5 μm wavelength range. For example, for a wavelength of 3.5 μm, we obtain *L_B_* ∼6.92 mm. Therefore, it is evident that waveguides with small birefringence require large cavity lengths in contrast with the general requirement of small chip area occupation. However, the cavity length has to be determined to simultaneously satisfy the condition *L_cavity_* ≫ *L_B_* and the condition able to induce SRS effect into the cavity.

**Figure 5.**
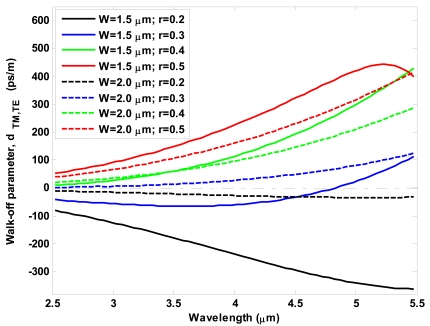
Walk-off parameter spectra for different values of *W* and *r*.

Another important optical characterization of SOI waveguides in mid-IR region can be made in terms of group velocity mismatch between quasi-TE and quasi-TM modes. Figure 5 shows the walk-off parameter defined as $d_{\text{TM},\text{TE}}=v_{g}^{-1}(\text{TM})-v_{g}^{-1}(\text{TE})$ versus operating wavelength, for different values of *W* and *r*. Similarly to simulations in Figure 4, the rib total height *H* has been assumed of 2.2 μm.

As a general trend, the spectral curves of Figure 5 show that the walk-off absolute value increases with increasing the wavelength. However, three cases are worthy to be pointed out. The first corresponds to the waveguide width *W*= 1.5 μm and *r* = 0.5, where an absolute maximum takes place around the operating wavelength of 5.22 μm. The second case is related to a SOI waveguide with *W* = 1.5 μm and *r* = 0.3, where a zero crossing occurs at a wavelength of 4.82 μm. In this case, it is evident how two optical pulses, polarized in orthogonal states and injected into the waveguide, will propagate with the same velocity. Finally, it seems to be very interesting the case for *W* = 2.0 μm and *r* = 0.2. For this waveguide, we can observe a flat shape around zero level in the wavelength range 2.5 ÷ 3.5 μm. It is important to outline that this last case induces both weak modal birefringence and very small group velocity mismatch between quasi-TE and quasi-TM modes.

### 3.3. Design Guidelines for Cascaded Raman Lasers

Recently, efficient Raman lasing in silicon has been demonstrated in NIR region [26,27], showing great potential for realizing low-cost, compact, room-temperature lasers even in MWIR region [31]. Such lasers are highly desirable for many applications ranging from trace-gas sensing, environmental monitoring and biomedical analysis, to industrial processes control and free-space communications. To the best of our knowledge, only a few papers are reported in literature on MWIR Raman lasers design. In fact, in the most of experimental and theoretical papers, as in [30,31], any comment about influence of waveguide size and mode polarization on the laser performance are not reported. Thus, the goal of this sub-section is to suggest a number of numerical simulations as main guidelines to design both laser optical waveguide and directional coupler, with the aim to optimize the lasing effect.

**Figure 6.**
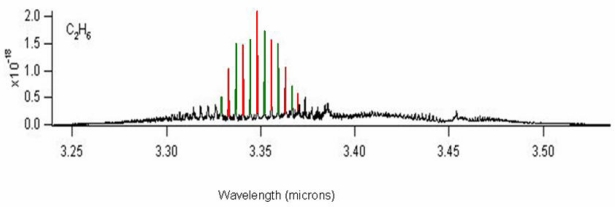
Ethane spectrum around 3.35 μm.

Without any loss of generality, hereinafter we focus our attention on the design of a Raman laser source for ethane gas sensing. In Figure 6 the ethane spectrum is sketched, characterized by an absorption peak centered at 3.3485 μm. Thus, we assume the first-order Stokes wavelength of *λ_s_*_1_ = 3.3485 μm for ethane gas detection. This implies a laser pump tuned at *λ_p_* = 2.8519 μm (antimony-based laser), while the higher-order Stokes waves are originated through cascaded Raman effect at *λ_s_*_2_ = 4.0544 μm and *λ_s_*_3_ = 5.1376 μm, respectively.

In the following analysis, we guess that the pump wave is aligned with quasi-TE polarized mode (as usually occurs in most experimental set-ups). Under this assumption, Equation (2) holds only for *ζ* = 1. In addition, since quasi-TM polarization is filtered out, the slowly-varying wave amplitude *A*_2_ is zero and then all terms in *k_i,j,k,l_* and proportional to *A*_2_ have to be set to zero in Equation (2). However, Equations (3)–(4) hold their validity because a quasi-TE pump pulse could in principle generate Stokes waves of both polarizations.

**Figure 7.**
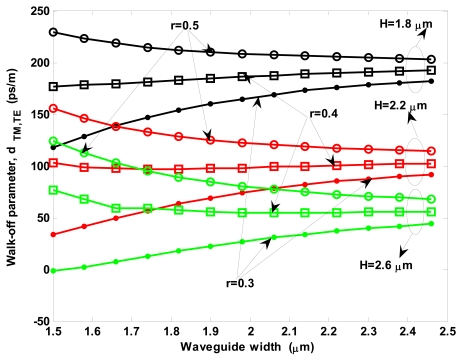
Walk-off parameter between first-order Stokes and pump waves for different values of *H* and *r* (*λ_p_* = 2.8519 μm, *λ*_s1_ = 3.3485 μm).

For the following discussion, we assume that the first-order Stokes wave grows-up being mainly aligned as a quasi-TM mode. This assumption will be verified in the next sub-section, due to smaller optical area and thus larger Raman modal gain of quasi-TM over quasi-TE mode. Under this assumption, we can evaluate as the walk-off parameter between quasi-TM first-order Stokes wave and quasi-TE pump is influenced by the waveguide sizes. Then, Figure 7 shows this walk-off parameter versus waveguide width, for different values of rib total height *H* and *r*.

As a general trend, Figure 7 shows the walk-off increase with decreasing *H* for any value of both *r* and waveguide width. Moreover, for each value of *H* the curves show a monotonically decreasing shape as a wavelength function in case of *r* = 0.5, while assuming the opposite trend for *r* = 0.3. In case of *r* = 0.4, the curve is monotonically decreasing or increasing for *H* = 2.6 μm and *H* = 1.8 μm, respectively. In addition, a very interesting case is represented for *r* = 0.3 and *H* = 2.6 μm, where a zero crossing at *W* = 1.5334 μm occurs. Thus, a waveguide designed with this optimal size can guarantee the rigorous absence of walk-off effect from a physical point of view, according to relation (8).

As demonstrated in [38], the walk-off effect could represent the limiting factor for pulsed Raman lasers. In fact, the walk-off length *L_w_* = *T*_0_/‖*d*_TM,TE_‖ could be shorter than the cavity length (*T*_0_ = *T_FWHM_*/1.665 for Gaussian pulses). Then, SRS is limited by the group-velocity mismatch and occurs only over distances *z*∼*L_w_*, even if the cavity length *L_cavity_* ≫ *L_w_*. At the same time, the nonlinear effects such as SPM and XPM become important because of the relatively large peak powers, considerably affecting the evolution of both pump and Raman waves. Thus, our optimal waveguide designed in this sub-section definitely avoids the walk-off problem in any case, including pulsed operation. Then, SRS effect induced in the resonant microcavity could be only limited by the enhancement factor of the pump wave.

**Figure 8.**
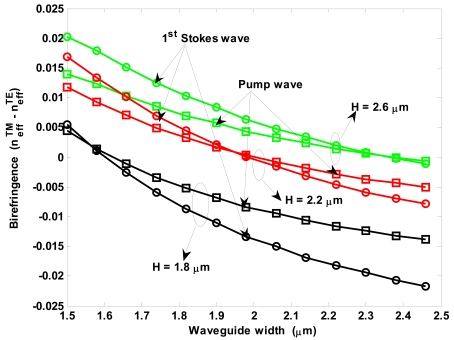
Modal birefringence for pump and first-order Stokes waves versus waveguide width for different values of *H* (*λ_p_* = 2.8519 μm, *λ_s_*_1_ = 3.3485 μm).

Figure 8 shows the modal birefringence for pump and first-order Stokes waves versus waveguide width, for different values of *H* and *r =* 0.3. The plot shows zero crossings for both pump and Stokes waves only for *H* < 2.2 μm. In particular, the birefringence free waveguide width decreases with decreasing *H*. The curves shown in Figure 8 can be used to estimate the beat length *L_B_*. In particular, for the optimal waveguide (*r* = 0.3, *H* = 2.6 μm and *W* = 1.5334 μm), we calculate *L_B_*(*pump*) = 214.42 μm and *L_B_*(1^st^ *Stokes*) = 174.4 μm.

Thus, an optical resonator with *L_cavity_* ≫ 214.42 μm has to be used to minimize the nonlinear birefringence effect. In this condition, it could be assumed that two Stokes waves of the same order but aligned in orthogonal polarizations are simultaneously amplified in a SOI waveguide by the same pump pulse, without any reciprocal interference. Their different behaviour will only depend on effective area and coupling factor.

**Figure 9.**
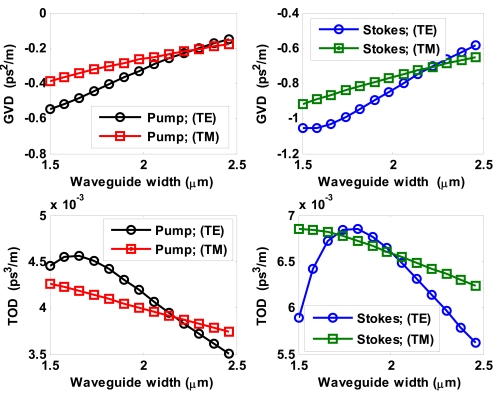
GVD and TOD coefficients versus waveguide width for pump and Stokes waves (*λ_p_* = 2.8519 μm, *λ_s_*_1_ = 3.3485 μm).

Further, we show in Figure 9 both GVD and TOD coefficients as a function of waveguide width for both pump and Stokes waves. In the simulations we have assumed *r* = 0.3, *H* = 2.6 μm and *W* = 1.5334 μm, and both polarizations. It is interesting to note how GVD coefficients assume a negative value for both pump and first-order Stokes waves and both polarizations. In addition, GVD and TOD coefficients are larger than those obtained in SOI waveguides operating in near-IR region [39], opening important challenges for nonlinear signal processing in mid-IR.

Now, both optical waveguides and minimum cavity length are designed and estimated. Another very important aspect is to find the design guidelines for the directional coupling. In fact, as shown by a formula derived in [38], the output efficiency and the lasing threshold for first-order Stokes depends strongly on the coupling factors related to the waves propagating inside the optical resonator. In particular, to reduce the threshold level for the first-order Stokes wave, it is essential to maximise the enhancement effect into the racetrack resonator. By defining the enhancement factor as Γ = ‖*A_ζ_*_,max_/*S_ζ_*‖, being *A_ζ_*_,max_ the pump amplitude maximum inside the cavity and *S_ζ_* the input pump peak outside the cavity for a fixed polarization state, the design of architecture in Figure 1 requires the condition Γ ≫ 1.

Due to the absence of detrimental effects induced by FCA and TPA contributions in mid-IR, it is possible to estimate *a priori*, with good approximation, the condition to maximise the enhancement factor. In fact, by considering a simple linear coupling mechanism and neglecting all nonlinear effects, it is possible to demonstrate that Γ factor depends essentially on the ratio *K* = *τ_l_*_,_*_ζ_*/*τ_c_*_,_*_ζ_* between total loss and pump coupling decay times. Following the standard conventions, under-coupling is denoted by *K* < 1, over-coupling by *K* > 1 and critical coupling by *K* = 1. This last condition means vanishing bus waveguide transmission, and thus it represents the optimal condition to maximise the pump energy inside the optical resonator, minimizing the Raman lasing threshold.

The previous guideline is rigorous if we are mainly focusing on first-order Stokes wave emission. In the case of cascaded Raman laser [31], it is generally appropriate to design the coupler close to the critical coupling for the pump wavelength; close to zero coupling for the first Stokes wavelength (achieving high intracavity power that generates Raman gain at the second-order Stokes wavelength) and low coupling for the second-order Stokes, in order to obtain at the same time both low lasing threshold and enough output power extraction from the resonator [31]. In our work, the design criteria for directional coupler are substantially different. In fact, with the aim to design a Raman laser for sensing of two different gases, polarization selectivity and output power extraction of the same order of magnitude for the first and second-order Stokes waves are required.

**Figure 10.**
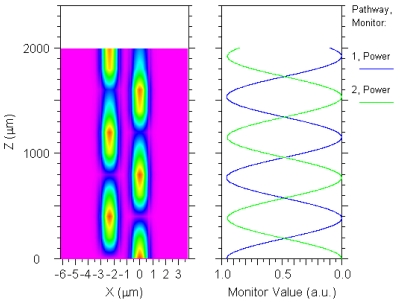
Power exchange in the directional coupler for pump wave (*λ_p_* = 2.8519 μm).

A number of 3D simulations based on BPM show a good trade-off between previous requirements when we consider a gap *G* = 0.8 μm and a coupling length *L_coup_* = 1,486 μm, being the coupling factors 3.13% and 2.47% for first and second-order quasi-TM Stokes waves, respectively. In turn, these values guarantee low threshold and similar output power extraction for both Stokes waves. Figure 10 shows the power exchange in the directional coupler versus coupling length for the pump wave tuned at 2.8519 μm and aligned as a quasi-TE mode, for *G* = 0.8 μm.

The plot also shows a quasi-TE pump wave coupling factor of 5% at *L_coup_* = 1,486 μm. Thus, assuming the total propagation loss inside the cavity $\alpha_{\text{loss}}^{\text{tot}}=5.0657{\text{m}}^{-1}(0.22\text{dB}/\text{cm})$ [39], the critical coupling condition for the pump $\left(\eta_{\xi }^{2}=L_{\text{cavity}}\alpha_{\text{loss}}^{\text{tot}}\right)$ requires a coupling factor ranging from 5.06% to 20.26% as the cavity length increases from 1 to 4 cm. Therefore, hereinafter *L_cavity_* = 1 cm is assumed. In Table 1 the coupling factors for Stokes waves in directional coupler are summarized.

Using the waveguide sizes and coupling factors, we can progress to the cascaded Raman laser simulation in the range 2.85 ÷ 4.0544 μm. According with some experimental works (i.e., [2,29,31]), we have considered in our numerical investigations silicon chip orientation [1 0 0] and Raman gain of *g_R_* = 9 cm/GW. However, to the best of our knowledge, experimental indications are not mentioned about any Raman gain anisotropy with respect to pump beam polarization. In addition, we assume *n*_2_ = 5 × 10 ^−5^ cm^2^/GW, *β^TPA^* ≃ 0 and negligible FCA effect [2].

The pump is assumed aligned as a quasi-TE mode with *n_eff_*_,1_ = 3.2911, *A_eff_*_,1_ = 2.4585 μm^2^ and ν*_g_*_,1_ = 8.3424 × 10^7^ m/s. The optical waveguide parameters, related to Stokes waves and obtained by means of full-vectorial FEM method [45], are summarized in Table 2.

To complete the cascaded Raman laser design, some comments about the absorption losses in the mid-IR region are needed. The total propagation loss inside the cavity $\alpha_{\text{loss}}^{\left(\text{tot}\right)}$ is strongly influenced by the material absorption losses that can become the dominant contribution in the mid-IR region. In fact, SOI technology in the mid-IR is usually limited by the silica that present an absorption loss coefficient less than 1dB/cm over 0.25–3.6 μm, except for a spike of 10 dB/cm in the range 2.6–2.9 μm [1].

Thus, according with results proposed in [1], the total absorption loss coefficient $\left(\alpha_{ab}^{\left(\text{tot}\right)}\right)$ for SOI rib waveguide can be estimated as:

(10) $$\alpha_{ab}^{\left(\text{tot}\right)}=\Gamma_{Si}\alpha_{Si}^{\left(\text{bulk}\right)}+\Gamma_{SiO_{2}}\alpha_{SiO_{2}}^{\left(\text{bulk}\right)}$$

being $\alpha_{Si}^{\left(\text{bulk}\right)}$ and $\alpha_{SiO_{2}}^{\left(\text{bulk}\right)}$ the bulk absorption of silicon and silica, respectively. The terms Γ*_Si_* and Γ*_SiO2_* are guided-power fractions into silicon and silica layer, respectively. Equation (10) suggests that it is possible to guarantee a modal low optical loss coefficient if SOI rib waveguide is designed with *H_s_* large enough to reduce the field tails inside the silicon oxide layer. As mentioned before, the critical wavelengths for the cascaded Raman laser as proposed in this work could be *λ_p_* = 2.8519 μm, and *λ_s_*_2_ =4.0544 μm, where silicon oxide presents large absorption values $\alpha_{SiO_{2}}^{\left(\text{bulk}\right)}$ ∼10dB/cm, and ∼7dB/cm, respectively. However, the silicon layer presents a very low absorption value, i.e., $\alpha_{Si}^{\left(\text{bulk}\right)}$ ∼ 0.001dB/cm for both wavelengths, and only a very small fraction of optical power is confined inside the silicon oxide. Thus, our estimation of $\alpha_{ab}^{\left(\text{tot}\right)}$ using Equation (10) and summarized in Table 3 demonstrate that the optimistic goal of $\alpha_{\text{loss}}^{\text{tot}}=0.22\text{dB}/\text{cm}$ can be really achieved with the SOI waveguide designed and proposed in this work.

**Figure 11.**
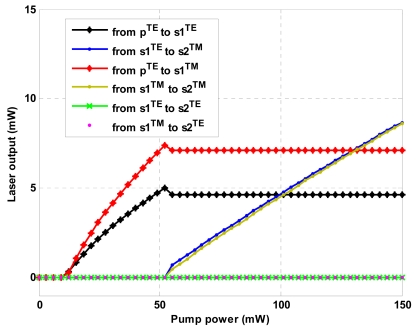
Laser output versus pump power for different combinations of polarization states.

Figure 11 shows the output of the cascaded Raman laser as designed and operating in continuous-wave regime. In our analysis, the laser output is calculated at the bus waveguide end as:

(11) $$P_{\text{out}}=\eta_{s}^{2}P_{s}e^{-\alpha_{\text{loss}}^{\text{tot}}L_{\text{Bus}}/2}$$

where *P_s_* is the power inside the cavity for the specific Stokes wave and *L_Bus_* represents the total bus length (see Figure 1).

In the plot we can observe the output power for first and second-order Stokes waves considering both polarizations. Several comments are worthy to give. First, the quasi-TE second-order Stokes wave presents a large value of lasing threshold, thus it cannot be lasing with an input power less than 150 mW. On the contrary, the quasi-TM second-order Stokes presents similar lasing threshold and external output efficiency, independently from the polarization state of exciting first order Stokes. It seems very interesting the first-order Stokes wave lasing. In fact, in this case the coupling factors for quasi-TE and quasi-TM polarizations do not represent a discriminating factor for the threshold level, since it has the same order of magnitude. In any case, above threshold (equal for both polarizations) the quasi-TM mode presents an output power level and an external efficiency larger than that for quasi-TE mode, as induced by smaller core area and larger coupling factor. Thus, the quasi-TM polarization is well suitable for the simultaneous lasing of the first two Stokes waves, allowing detection of two gases.

**Figure 12.**
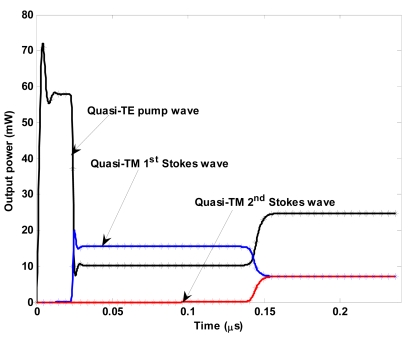
Time dynamics for both pump and Stokes waves (*λ_p_* = 2.8519 μm, *λ_s_*_1_ = 3.3485 μm, *λ_s_*_2_ = 4.0544 μm).

Moreover, Figure 11 demonstrates an output power of 7.136 mW for both first and second-order Stokes waves for an input power of 131.25 mW. Under this condition, the time dynamics for the designed cascaded Raman laser is sketched in Figure 12. It is worth to note as the time reference moves with the waves, as indicated by relationship (9), and the designed cascaded Raman laser operates in continuous-wave regime. The previous simulations demonstrate that not only the cascaded Raman lasing can be achieved in SOI waveguides working at mid-IR, but also that its performance are well suitable for gas sensing environmental applications in terms of reduced power consumption, stability, wavelength tunability and polarization selectivity. In addition, our theoretical investigations have demonstrated the possibility to obtain output powers larger than 7 mW, much better than in other approaches, i.e., 0.2 mW as mentioned in [3], with consequent positive impact on relevant electronic signal processing and device sensitivity.

In particular, the Raman laser is designed to detect two different types of gases, i.e., C_2_H_6_ and CO_2_ [3]. It is evident that, in spite of an increasing input pump power, the third-order Stokes wave lasing at *λ_s_*_3_ = 5.1376 μm could be also obtained, giving for example the possibility to detect a third gas, such as NO [3].

## 4. Conclusions

The generalized model presented in this paper allows to accurately predict the time dynamics of pump and Stokes waves in cascaded Raman lasers based on a SOI microcavity resonator and operating in mid-IR region. In addition, the theoretical study has allowed to demonstrate high performance of cascaded Raman laser in terms of reduced power consumption, stability, wavelength tunability and polarizations selectivity, offering unique advantages as mid-IR optical sources and thus competing with complex and bulk solid-state laser systems. Finally, a detailed design of a cascaded Raman laser for gas sensing of C_2_H_6_ and CO_2_ has been developed under CW operation. This approach could be also used for gas sensing at longer mid-IR wavelengths with improved sensitivity.

## Figures and Tables

**Table 1.** Coupling factors for Stokes waves.

	**η^2^ (quasi-TE) (%)**	**η^2^ (quasi-TM) (%)**
1^st^ Stokes wave (*λ_s_*_1_ = 3.3485 μm)	1.81	3.13
2^nd^ Stokes wave (*λ_s_*_2_ =4.0544 μm)	65.14	2.47

**Table 2.** Optical waveguide parameters for Raman laser simulations.

	**Quasi-TE modes**			**Quasi-TM modes**		

	***n_eff_***	***A_eff_* (μm^2^)**	***v_g_* (m/s)**	***n_eff_***	***A_eff_* (μm^2^)**	***v_g_* (m/s)**
1^st^ Stokes wave (λ*_s_*_1_ = 3.3485 μm)	3.2363	2.6289	8.2892×10^7^	3.2555	2.3418	8.3424×10^7^
2^nd^ Stokes wave (λ*_s_*_2_ = 4.0544 μm)	3.1518	2.9302	8.1974×10^7^	3.1802	2.4776	8.2537×10^7^
3^rd^ Stokes wave (λ*_s_*_3_ = 5.1376 μm)	3.0088	3.5665	8.0725×10^7^	3.0497	2.7383	8.0812×10^7^

**Table 3.** Total absorption coefficients.

	**Quasi-TE modes**			**Quasi-TM modes**		

	Γ*_Si_*	Γ*_SiO2_*	**(dB/cm)**	Γ*_Si_*	Γ*_SiO2_*	**(dB/cm)**
Pump wave (*λ_p_* =2.8519 μm)	0.9917	0.0041	0.042	0.9910	4.95×10^-4^	0.0059
2^nd^ Stokes wave (*λ_s2_* = 4.0544 μm)	0.9731	0.0137	0.096	0.9776	0.0015	0.011
